# Positive results for patients with COVID-19 discharged form hospital in Chongqing, China

**DOI:** 10.1186/s12879-020-05151-y

**Published:** 2020-06-19

**Authors:** Wang Deng, Tian-wen Guang, Mei Yang, Jian-rong Li, De-peng Jiang, Chang-yi Li, Dao-xin Wang

**Affiliations:** 1 Department of Respiratory and Critical Care Medicine, Second Affiliated Hospital of Chongqing Medical University, 76 Linjiang Road, Yuzhong District, Chongqing, 400010 China; 2 Chongqing Medical Research Center for Respiratory and Critical Care Medicine, Chongqing, 400010 China; 3 Department of Infectious Disease, Yongchuan Affiliated Hospital of Chongqing Medical University, 439 Xuanhua Road, Yongchuan District, Chongqing, 402160 China; 4 Department of Respiratory Medicine, Chongqing Public Health Medical Center, 109 Baoyu Road, Shapingba District, Chongqing, 400036 China; 5 Department of Respiratory Medicine, Wanzhou General Hospital, 27 Guoben Road, Wanzhou District, Chongqing, 404100 China

**Keywords:** Coronavirus disease 2019, Hospital discharge, SARS-CoV-2

## Abstract

**Background:**

Since December 2019, over 80,000 patients with coronavirus disease 2019 (COVID-19) have been confirmed in China. With the increasing number of recovered patients, more attention should be paid to the follow-up of these patients.

**Methods:**

In the study, 576 patients with COVID-19 discharged from hospital in Chongqing, China from January 24, 2020, to March 10, 2020 were evaluated by viral nucleic acid tests for severe acute respiratory syndrome coronavirus 2(SARS-CoV-2) to determine if they could be released from quarantine. Among the 576 patients, 61 patients (10.6%) had positive RT-PCR test results of SARS-CoV-2. We aimed to analyze the demographics, clinical characteristics and treatment of 61 patients.

**Results:**

These positive patients were characterized by older age, chronic medical illness and mild conditions. 38 (62.3%) patients who were asymptomatic without abnormalities on chest radiographs were found in the positive with COVID-19. Also, they showed positive results of stool or sputum specimens with negative results of nasal and pharyngeal swab specimens. The median duration of positive result of SARS-CoV-2 was varied from 3 days to 35 days in the patients discharged from hospital with no family member infection.

**Conclusions:**

Multi-site screening of SARS-CoV-2 including nasal and pharyngeal swabs, stool and sputum specimens could be considered to improve the diagnosis, treatment and infection control in patients with COVID-19. Our findings provide the important information and clinical evidence for the improved management of patients recovered from COVID-19.

## Background

Since December 2019, an unknown pneumonia broke out in Wuhan, Hubei Province, China, which has rapidly spread from China to at least 200 countries abroad. A novel coronavirus disease 2019 (COVID-19) was identified with the similar clinical manifestations to severe acute respiratory syndrome (SARS) and Middle East respiratory syndrome (MERS) [1]. COVID-19 was issued a global outbreak and pandemic by World Health Organization [2] with over 3,000,000 confirmed cases by April 29, 2020.So far, more than 80,000 patients with COVID-19 has been confirmed in China. Elderly patients with co-morbidities were prone to develop the high risk for severe and critically ill situation and require intensive care medical interventions [3, 4]. With the increasing number of recovered patients discharged from hospital by regular follow-up and medical observation, some medical professionals were found to be positive for COVID-19 after hospital discharge [5], and more attention should be paid to the follow-up of non- medical patients.

Severe acute respiratory syndrome coronavirus 2(SARS-CoV-2) can be detected in the specimens of upper respiratory tract, stool and lower respiratory tract [6]. Currently, a small number of positive results of SARS-CoV-2 in some recovered patients have been reported [1, 7, 8], but the management of these positive patients still remains an unsolved problem. In addition, there is a lack of clinical characteristics, the potential impact and significance of positive patients recovered from COVID-19, which makes it difficult to provide clinical evidence and experience for the management of patients with COVID-19 in the recovery period.

The objectives of this study were to investigate the clinical characteristics of patients discharged form hospital with positive results of SARS-CoV-2. In the study, 576 patients with COVID-19 discharged from hospital were retrospectively analyzed in Chongqing, China. Among them, 61 patients had positive results of SARS-CoV-2 by real-time reverse transcriptase polymerase chain reaction (RT-PCR) test, which provided the important information and clinical evidence for the improved management of patients recovered from COVID-19.

## Methods

Five hundred seventy-six patients with COVID-19 discharged from Chongqing Public Health Medical Center, Yongchuan Affiliated Hospital of Chongqing Medical University, and Wanzhou General Hospital, Chongqing, China, from January 24, 2020, to March 10, 2020 were evaluated by RT-PCR assay for SARS-CoV-2 according to the manufacturer’s protocol (Jiangsu Perfectus Biotechnology Co., Ltd., patch No. JC10223-1 N) to determine if they could be released from quarantine at home. Recovered patients discharged from hospital or discontinuation of quarantine should meet the following criteria [9]: (1) normal temperature for more than 3 days, (2) significant improvement of respiratory symptoms, (3) significant absorbtion of acute exudative lesions on chest radiograph and (4) two consecutively negative results by RT-PCR assay of nasal and pharyngeal swabs with at least 1 day interval.

We extracted the clinical symptoms information, laboratory findings and radiologic abnormality form electronic medical records. Patients were recommended quarantine at home after hospital discharge and returned to hospital for viral nucleic acid detection by RT-PCR. Nasal and pharyngeal swab specimens were collected as previously described [10]. Stool and sputum specimens were also collected for viral nucleic acid detection by RT-PCR. Two consecutively tests by RT-PCR of nasal and pharyngeal swab specimens, stool and sputum specimens were performed with at least 1 day interval combined with chest radiograph during the quarantine period.

Continuous variables were presented as mean (SD) or median (IQR) and categorical variables as count (%).SPSS (version 13.0) was used for all analysis.

The study was approved by the Ethics Committee of the Second Affiliated Hospital of Chongqing Medical University and the need for informed consent was waived. The patients have not been reported in any other submission by anyone else.

## Results

Among the patients discharged from hospital, 61 (10.6%) patients had positive results of SARS-CoV-2 by RT-PCR tests. None of the patients were medical professionals. Demographics, clinical characteristics and treatment of 61 patients were shown in Table 1. The median age of 61 patients was 54.79 years, including 36(59%) female patients and 25 (41%) male patients. 24 (39.3%) patients had chronic diseases, including chronic obstructive pulmonary disease, hypertension, diabetes, digestive system disease, cerebrovascular disease, chronic kidney disease and chronic hepatitis. 38 (62.3%) patients were asymptomatic. The most common symptoms were fever (24.6%), cough (18%), sputum production (14.8%), and sore throat (13.1%), while the less common symptoms were headache (8.2%), shortness of breath (4.9%), fatigue (4.9%) and diarrhea (4.9%). On admission, leucocytes were above the normal range in 8 (13.1%) patients and below the normal range in 11 (18%) patients.14 (22.9%) patients had neutrophils above the normal range. Lymphocytes and platelets were below the normal range in 17 (27.9%) patients and 4(6.6%) patients respectively. Nine patients had different degrees of liver function abnormality, with the increase in alanine aminotransferase or aspartate aminotransferase (Table 1). Most patients (95.1%) had hypoproteinemia. Fourteen patients had different degrees of renal function damage, with the elevation of blood urea nitrogen or serum creatinine. Chest computed tomography (CT) scan of symptomatic patients showed local patchy shadowing (14.8%), ground-glass opacity (4.9%), bilateral patchy shadowing (13.1%) and interstitial abnormalities (4.9%). Normal CT imaging was showed in asymptomatic patients. The severity of disease was mild status in 38(62.3%) patients.

**Table 1:** Demographics, clinical characteristics and treatment of patients recovered from COVID-19 with positive results of SARS-CoV-2

	Patients (*n* = 61)
**Age, years**	54.79 (12.89)
≤ 29	3 (4.9%)
30–39	7 (11.5%)
40–49	12 (19.7%)
50–59	18 (29.5%)
60–69	15 (24.6%)
70–79	6 (9.8%)
**Sex**	
Female	36 (59%)
Male	25 (41%)
**Chronic medical illness**	24 (39.3%)
Chronic obstructive pulmonary disease	8 (13.1%)
Hypertension	5 (8.2%)
Diabetes	5 (8.2%)
Digestive system disease	2 (3.3%)
Cerebrovascular disease	1 (1.6%)
Chronic kidney disease	1 (1.6%)
Chronic hepatitis	2 (3.3%)
**Symptoms after discharge from hospital**	
Fever	15 (24.6%)
Sore throat	8 (13.1%)
Cough	11 (18%)
Shortness of breath	3 (4.9%)
Sputum production	9 (14.8%)
Headache	5 (8.2%)
Fatigue	3 (4.9%)
Diarrhoea	3 (4.9%)
Asymptom	38 (62.3%)
**Positive results**	
Nasal and pharyngeal swab	36 (59%)
Stool	17 (27.9%)
Sputum	8 (13.1%)
**Disease severity status**	
Mild	38 (62.3%)
General	20 (32.8%)
Severe	3 (4.9%)
Critical	0
**Laboratory findings**	
Leucocyte count (× 10^9^ per L; normal range 3.5–9.5)	6.9 (4.5–8.5)
> 9.5	8 (13.1%)
< 3.5	11 (18%)
Neutrophil count (× 10^9^ per L; normal range 1.8–6.3)	4.9 (3.4–5.9)
> 6.3	14 (22.9%)
Lymphocyte count (× 10^9^ per L; normal range 1.1–3.2)	1.5 (0.8–1.9)
< 0·8	17 (27.9%)
Platelets count (× 10^9^ per L; normal range 100–300)	155 (128–175)
< 100	4 (6.6%)
Procalcitonin, ng/mL (normal range < 0.05)	
≥ 0.05, n (%)	6 (9.8%)
C-reactive protein (mg/L; normal range 0–5)	3.2 (1.7–28.6)
≥ 5.0	19 (31.1%)
D-dimer (mg/L; normal range 0–500)	164 (65–327)
> 500	7 (11.5%)
Albumin (g/L; normal range 40–55)	33.7 (28.4–37.6)
< 40	58 (95.1%)
Alanine aminotransferase (U/L; normal range 9–50)	27 (19–36)
> 50	5 (8.2%)
Aspartate aminotransferase (U/L; normal range15–40)	24 (15–33)
> 40	4 (6.6%)
Blood urea nitrogen (mmol/L; normal range3.6–9.5)	6.1 (4.7–7.3)
> 9.5	5 (8.2%)
Serum creatinine (μmol/L; normal range 57–111)	80 (67–95)
> 111	9 (14.8%)
Lactate dehydrogenase (U/L; normal range 120–250)	172 (150–262)
> 250	14 (22.9%)
**Radiologic findings**	
Abnormalities on chest radiograph	23 (37.7%)
Ground-glass opacity	3 (4.9%)
Local patchy shadowing	9 (14.8%)
Bilateral patchy shadowing	8 (13.1%)
Interstitial abnormalities	3 (4.9%)
Normalities on chest radiograph	38 (62.3%)
**Length of stay in hospital, days**	18.0 (14.0–22.5)
**Length of positive result for COVID-19 after hospital discharge, days**	10 (7–13)
< 14	47 (77%)
14–28	9 (14.8%)
> 28	5 (8.2%)
**Treatment**	
Oxygen therapy	21 (34.4%)
Non-invasive mechanical ventilation	2 (3.3%)
Antiviral treatment	23 (37.7%)
Antibiotic treatment	20 (32.8%)
Glucocorticoids	2 (3.3%)
Convalescent plasma therapy	3 (4.9%)
**Clinical outcomes at data cutoff**	
Hospitalization	22 (36.1%)
Hospital Discharge	1 (1.6%)
Centralized isolation for medical observation	38 (62.3%)

Data are n (%), mean (SD) and median (IQR).OVID-19: coronavirus disease 2019, SARS-CoV-2: severe acute respiratory syndrome coronavirus 2

Sixty-one patients were received treatment for 18 days (IQR 14.0–22.5) and had two consecutively negative results of nasal and pharyngeal swab specimens by RT-PCR before discharge. They were required to continue the quarantine at home for at least 14 days. During the isolation period, 36 (59%) patients had positive results of SARS-CoV-2 by nasal and pharyngeal swabs;17(27.9%) patients had positive results of SARS-CoV-2 by stool; 8 (13.1%) patients had positive results of SARS-CoV-2 by sputum. From the day of hospital discharge, 47(77%) patients had positive results of SARS-CoV-2 for less than 14 days, whereas 14(23%) patients had positive results for more than 14 days (Table 1). The median duration of positive RT-PCR test results of SARS-CoV-2 was 10 days (IQR 7–13) in 61 patients discharged from hospital with the shortest length of 3 days and longest length of 35 days. They had no contact with any person presenting respiratory symptoms. No family member infection was found.

23(37.7%) patients were administered antiviral therapy (lopinavir/ritonavir) and 20 (32.8%) patients received empirical antibiotic treatment. 2 (3.3%) patients were given systematic corticosteroids. 3 (4.9%) patients were given convalescent plasma therapy. By March 10, 2020, 22 (36.1%) patients were remained in hospital. 1 (1.6%) patients was discharged and 38 (62.3%) patients with no symptoms were maintained isolation for medical observation before negative results of viral nucleic acid detection.

## Discussion

Currently, the diagnosis of SARS-CoV-2 is dependent on viral nucleic acid detection. Sixty-one patients with COVID-19 had positive results by RT-PCR that fulfilled the criteria for hospital discharge during or more than the 14-day quarantine period. The underlying mechanisms of positive results of SARS-CoV-2 in recovered patients with COVID-19 remain unclear. The major factors including the different sampling tissues [11], false negative of RT-PCR test [12], immunological status [13], viral load and intermittent shedding [14], and viral distribution [15] are currently considered for the possible reasons of re-detectable positive. In the study, though both ORF1b gene and N gene of SARS-CoV-2 were detected using commercial kit, false-negative of test kit may partially account for the reason as previously reported [16]. According to our results, the rate of false-negative in virus detection was lower than a recent study reported by Xiao et al [17]. We found most recovered patients had hypoproteinemia, suggesting nutritional status probably involved for the positive results.

In the study, most patients (88.9%) with sputum production had positive results of SARS-CoV-2 after discharge. In addition, the median duration of positive result of SARS-CoV-2 was varied from 3 days to 35 days after hospital discharge, suggesting the intermittent shedding of virus might occur in recovered patients. Fourteen patients had positive results of stool specimens for more than 14 days with negative results of nasal and pharyngeal swabs, suggesting viral shedding from the digestive system lasting longer than that from the respiratory tract. Therefore, stool or sputum specimen-testing might be benefit for the detection of SARS-CoV-2 in determining the diagnosis, treatment and termination of quarantine [18, 19]. Positive results of SARS-CoV-2 determined by stool and sputum also indicated that viral distribution in different sampling tissues and multiple shedding routes [20, 21]. Positive results occurred in most recovered patients with COVID-19 might not be caused by virus recurrence or second virus infection. Sampling tissues of multiple sites could be considered for recovered patients with COVID-19. In this study, patients with two consecutively negative results of nasal and pharyngeal swabs were shown positive results of stool or sputum specimens using RT-PCR test after discharge, indicating the necessity of stool and sputum specimens by RT-PCR adding to the criteria for discharge or discontinuation of quarantine.

The positive rate of specimen detection is limited by the level of viral nucleic acid [11]. The detection of virus RNA was dependent on viral load, suggesting the potential SARS-CoV-2 replication in different sampling tissues [22]. Viral nucleic acid was detected by ORF1b gene and N gene of SARS-CoV-2 in the study. It was difficult for RT-PCR to distinguish the viral activity. Despite the compliance with discharge criteria, viral RNA remained positive in nasal and pharyngeal swabs, stool and sputum specimens in the recovered patients with COVID-19. Viral residual and delay in clearance of viral RNA might be considered as the potential factors [7]. Further studies should address isolation of SARS-CoV-2 in tissue specimens of recovered patients to identify the viral activity. Also, the IgM-IgG combined assay in blood samples could be considered for the potentially rapid screening of SARS-CoV-2 infection in patients [23].

For a small proportion of patients who had positive results of SARS-CoV-2 for more than 14 days, virus carrier status probably existed. Appropriate prolongation of isolation period should be also further proposed. The transmissibility of COVID-19 is mainly dependent on the high level of SARS-CoV-2 shedding in the upper respiratory tract, even among presymptomatic patients [24]. More than half of residents with positive results of SARS-CoV-2 were asymptomatic in a skilled nursing facility reported by Arons et al. [25] The viral load of asymptomatic patients with positive results of SARS-CoV-2 was similar to that in symptomatic patients, indicating the potential transmission [26]. Therefore, asymptomatic infection may play an important role in the spread of SARS-CoV-2.In our study, the screening protocol of asymptomatic patients during quarantine period for viral nucleic acid detection was necessary for management and control transmission. Surprisingly, non-infected family members were reported during home quarantine in the study. The management of asymptomatic patients with COVID-19 requires further investigation. Patients with symptoms, abnormalities on chest radiograph and abnormal laboratory results were received antiviral treatment in time. Convalescent plasma therapy was performed in severe patients.

The study has a limitation of small sample of patients with COVID-19 discharged form hospital. Also, the lack of serum-specific antibody levels testing in the recovered patients with COVID-19 was due to the shortage of testing load and medical resource and clinical workload in the frontline during the outbreak. A larger cohort study is necessary to investigate the prognosis and transmission risk of recovered patients with COVID-19.

## Conclusions

The study revealed the clinical features of recovered patients with the recurrence of positive results of SARS-CoV-2.Multi-site screening including nasal and pharyngeal swabs, stool and sputum specimens could be considered to improve the diagnosis, treatment and infection control in patients with COVID-19. These findings provide the important information and clinical evidence for the management of recovered patients with COVID-19. Criteria for hospital discharge or discontinuation of quarantine would be updated with the progress of clinical evidence and experience accumulation.

## Data Availability

All data analyzed during this study are included in this published article. The raw datasets used for the analysis are available from the corresponding author on reasonable request.

## Abbreviations

COVID-19: Coronavirus disease 2019
CT: Computed tomography
MERS: Middle East respiratory syndrome
RT-PCR: Real-time reverse transcriptase–polymerase chain reaction
SARS-CoV-2: Severe acute respiratory syndrome coronavirus 2
SARS: Severe acute respiratory syndrome
